# Natural history, outcome measures and trial readiness in LAMA2-related muscular dystrophy and SELENON-related myopathy in children and adults: protocol of the LAST STRONG study

**DOI:** 10.1186/s12883-021-02336-z

**Published:** 2021-08-12

**Authors:** Karlijn Bouman, Jan T. Groothuis, Jonne Doorduin, Nens van Alfen, Floris E. A. Udink ten Cate, Frederik M. A. van den Heuvel, Robin Nijveldt, Willem C. M. van Tilburg, Stan C. F. M. Buckens, Anne T. M. Dittrich, Jos M. T. Draaisma, Mirian C. H. Janssen, Erik-Jan Kamsteeg, Esmee S. B. van Kleef, Saskia Koene, Jan A. M. Smeitink, Benno Küsters, Florence H. J. van Tienen, Hubert J. M. Smeets, Baziel G. M. van Engelen, Corrie E. Erasmus, Nicol C. Voermans

**Affiliations:** 1 Department of Neurology, Donders Institute for Brain, Cognition and Behaviour, Radboud university medical center, Nijmegen, The Netherlands; 2 Department of Pediatric Neurology, Donders Institute for Brain, Cognition and Behaviour, Amalia Children’s Hospital, Radboud university medical center, Nijmegen, The Netherlands; 3 Department of Rehabilitation, Donders Institute for Brain, Cognition and Behaviour, Radboud university medical center, Nijmegen, The Netherlands; 4 Department of Pediatric cardiology, Amalia Children’s Hospital, Radboud university medical center, Nijmegen, The Netherlands; 5 Department of Cardiology, Radboud university medical center, Nijmegen, The Netherlands; 6 Department of Radiology, Radboud university medical center, Nijmegen, The Netherlands; 7 Department of Pediatrics, Amalia Children’s Hospital, Radboud university medical center, Nijmegen, The Netherlands; 8 Department of Internal Medicine, Radboud university medical center, Nijmegen, The Netherlands; 9 Department of Human Genetics, Radboud university medical center, Nijmegen, The Netherlands; 10 Department of Clinical Genetics, Leiden University Medical Center, Leiden, The Netherlands; 11 Khondrion BV, Nijmegen, The Netherlands; 12 Department of Pathology, Radboud university medical center, Nijmegen, The Netherlands; 13 Department of Toxicogenomics, Maastricht University, Maastricht, The Netherlands; 14 School for Mental Health and Neurosciences (MHeNS), Maastricht University, Maastricht, the Netherlands; 15 School for Developmental Biology and Oncology (GROW), Maastricht University, Maastricht, The Netherlands

**Keywords:** *LAMA2*, Laminin subunit α2 deficiency, Merosin-deficient congenital muscular dystrophy type 1A (MDC1A), SELENON, SEPN1, Natural history, Outcome measures, Trial readiness, All ages

## Abstract

**Background:**

SELENON (SEPN1)-related myopathy (SELENON-RM) is a rare congenital myopathy characterized by slowly progressive proximal muscle weakness, early onset spine rigidity and respiratory insufficiency. A muscular dystrophy caused by mutations in the *LAMA2* gene (LAMA2-related muscular dystrophy, LAMA2-MD) has a similar clinical phenotype, with either a severe, early-onset due to complete Laminin subunit α2 deficiency (merosin-deficient congenital muscular dystrophy type 1A (MDC1A)), or a mild, childhood- or adult-onset due to partial Laminin subunit α2 deficiency. For both muscle diseases, no curative treatment options exist, yet promising preclinical studies are ongoing. Currently, there is a paucity on natural history data and appropriate clinical and functional outcome measures are needed to reach trial readiness.

**Methods:**

LAST STRONG is a natural history study in Dutch-speaking patients of all ages diagnosed with SELENON-RM or LAMA2-MD, starting August 2020. Patients have four visits at our hospital over a period of 1.5 year. At all visits, they undergo standardized neurological examination, hand-held dynamometry (age ≥ 5 years), functional measurements, questionnaires (patient report and/or parent proxy; age ≥ 2 years), muscle ultrasound including diaphragm, pulmonary function tests (spirometry, maximal inspiratory and expiratory pressure, sniff nasal inspiratory pressure; age ≥ 5 years), and accelerometry for 8 days (age ≥ 2 years); at visit one and three, they undergo cardiac evaluation (electrocardiogram, echocardiography; age ≥ 2 years), spine X-ray (age ≥ 2 years), dual-energy X-ray absorptiometry (DEXA-)scan (age ≥ 2 years) and full body magnetic resonance imaging (MRI) (age ≥ 10 years). All examinations are adapted to the patient’s age and functional abilities. Correlation between key parameters within and between subsequent visits will be assessed.

**Discussion:**

Our study will describe the natural history of patients diagnosed with SELENON-RM or LAMA2-MD, enabling us to select relevant clinical and functional outcome measures for reaching clinical trial-readiness. Moreover, our detailed description (deep phenotyping) of the clinical features will optimize clinical management and will establish a well-characterized baseline cohort for prospective follow-up.

**Conclusion:**

Our natural history study is an essential step for reaching trial readiness in SELENON-RM and LAMA2-MD.

**Trial registration:**

This study has been approved by medical ethical reviewing committee Region Arnhem-Nijmegen (NL64269.091.17, 2017–3911) and is registered at ClinicalTrial.gov (NCT04478981).

## Background

Selenoprotein N-related congenital myopathy (SEPN1- or SELENON-RM) is a rare congenital myopathy with an estimated prevalence of 0.5 in 1000,000 [1]. Core features include slowly progressive axial muscle weakness, early-onset rigidity of the spine, scoliosis and respiratory insufficiency. Delayed motor development is the most common presenting sign. Muscle biopsies show multi-minicores as the most common lesion, often associated with mild dystrophic features [2]. Laminin α2-related muscular dystrophy (LAMA2-MD) has a similar clinical phenotype, with an estimated prevalence of 4 in 500,000 [3]. It has a heterogeneous disease spectrum ranging from a severe, early-onset congenital muscular dystrophy (complete Laminin subunit α2 deficiency, also called merosin-deficient congenital muscular dystrophy type 1A (MDC1A)) to a mild, childhood- or adult-onset limb-girdle type muscular dystrophy (partial Laminin subunit α2 deficiency). Additionally, patients may suffer from epileptic seizures and may show characteristic diffuse brain white matter lesions on magnetic resonance imaging (MRI) [4]. The clinical diagnosis of SELENON-RM and LAMA2-MD is confirmed by recessive pathogenic variants in the *SELENON* gene (OMIM-number 606210) or *LAMA2* gene (OMIM-number 156225), respectively [5–9]. Currently, no curative treatment options exist for neither SELENON-RM nor LAMA2-MD. Optimal pulmonary, cardiac, nutrition and orthopedic management, in combination with supportive care from rehabilitation and allied health care, is essential for preventing or treating severe complications [10, 11]. Further, promising new therapies are currently being developed [12–21].

Selenoprotein N is an endoplasmic reticulum (ER) calcium sensor that responds to diminished luminal calcium levels by refilling the ER calcium stores [22]. SELENON-RM has striking similarities at the cellular level with classical mitochondrial diseases. This has led to the hypothesis that sonlicromanol, a new clinical stage chemical entity with a dual activity as antioxidant and redox modulator developed for mitochondrial oxidative phosphorylation disturbances, is also beneficial for patients with SELENON-RM [12, 13]. Interestingly, the first results of experiments in an animal model (SELENON knock-out zebrafish) showed improved muscular function (unpublished data). Further, other antioxidants are also hypothesized to be beneficial in the treatment of patients with SELENON-RM [13–15]. In general, the recognition of the interplay between mitochondrial bioenergetics and endoplasmic reticulum paves the way to the identification of potential treatment options [16, 17]. Laminin subunit α2 is an extracellular matrix protein that links with dystrophin on the inner side of the muscle membrane. This linkage is of high importance for normal skeletal muscle function as it stabilizes the sarcolemma and protects the muscle fibers from contraction-induced damage [23, 24]. Further, a metabolic impairment, with reduced mitochondrial respiration and enhanced glycolysis, was observed in human Laminin subunit α2 deficient muscle cells [25]. For LAMA2-MD, preclinical studies on the use of linker proteins, on exogenous administration of Laminin-111, on upregulation of LAMA1, on genome editing technology and on the use of antioxidant molecules are being performed in animal models [18–21, 26, 27]. Moreover, different research groups are working on revealing the precise pathophysiology, which could eventually help to design new treatment strategies for SELENON-RM [14, 28–31] or LAMA2-MD [32–34].

In order to pave the way towards clinical trials, it is essential to identify and characterize the patients clinically and genetically, and to select clinical and functional outcome measures that correlate with muscle function and that are sensitive to change over time. These outcome measures can be used to determine the effectivity of possible treatment options in clinical trials. Three large clinical studies have recently been performed, one in SELENON-RM and two in LAMA2-MD. We discuss the main findings below.

In SELENON-RM patients, a retrospective clinical, histologic and genetic analysis of 132 pediatric and adult patients (age range at last examination 2 to 58 years) was performed by Villar-Quiles et al. [2]. The main prognostic determinants for disease severity included scoliosis and respiratory management, body mass abnormalities and the specific *SELENON* mutation found in the patient. The latter indicates a genotype-phenotype association between bi-allelic null mutations and more severe disease. This study reports the largest SELENON-RM series and the first one including pediatric, adolescent and adult patients followed-up for several decades. Limitations of this study are its retrospective design, and the absence of functional measurements and convenient muscle visualizing techniques (i.e. muscle ultrasound or MRI) performed in a standardized manner.

In LAMA2-MD patients, a 5-year prospective natural history study that included 24 patients (age range 4 to 22 years) was performed by Jain et al. [35, 36]. The MFM-32 was found to be sensitive to change in ambulatory and non-ambulatory patients with LAMA2-MD. In non-ambulatory patients, they found a yearly decline in knee flexion strength and passive range of motion (PROM) of left elbow extension. Limitations of this study included the small subset of examinations performed and the absence of convenient muscle visualizing techniques. Further, patients were selected based on a convenience sample, possibly leading to a selection bias. Moreover, the age range limits the availability on natural history data in the very young children (< 4 years) and in older adult patients (> 22 years). Recently, Zambon et al. published a retrospective longitudinal study on 46 patients with LAMA2-MD of whom 42 patients had a complete and 4 patients had a partial Laminin subunit α2 deficiency (age range at last examination 12 to 22 years) [37]. They found a linear decrease in passive range of motion of left elbow extension and a linear decline in percentage predicted forced vital capacity. The intrinsic limitations included the retrospective nature of data collection, the limited age range and the inconsistencies in the use of functional scales throughout follow-up (i.e. frequency of examinations, indication for ancillary examinations etcetera).

In short, a prospective natural history study in an unselected group of patients including a plethora of clinical and functional outcome measures is lacking in both SELENON-RM and LAMA2-MD. Due to the promising ongoing preclinical studies, there is a high need to obtain natural history data in order to reach trial readiness for both muscle diseases. The similarities in the clinical phenotype of both muscle diseases allows us to combine both studies in one study protocol.

## Objectives

The primary objectives of this study in order to reach trials readiness, are:

1. to assess 1.5-year natural history in patients with SELENON-RM or LAMA2-MD;
2. to select relevant and sensitive clinical and functional outcome measures.

The secondary objectives of this study are:

3. to provide prevalence estimations of SELENON-RM and LAMA2-MD in the Netherlands and Flanders (Dutch-speaking part of Belgium);
4. to establish a well-characterized baseline cohort of patients with SELENON-RM or LAMA2-MD for prospective follow-up and recruitment for future clinical trials;
5. to assess the clinical features to optimize clinical management for patients with SELENON-RM or LAMA2-MD.

## Methods / design

### Study design

Our study on LAMA2-MD and SELENON-RM To Study Trial Readiness, Outcome measures and Natural history (LAST STRONG) is a prospective, single-center, observational study with repeated measurements performed at the Department of Neurology and Pediatric Neurology within the neuromuscular center of the Radboud university medical center, The Netherlands. Our center is a tertiary referral center for neuromuscular diseases. Participation in the study will not affect the usual care provided by the patient’s own medical team. Patients are invited to visit our hospital four times over a period of 1.5 year, with an interval of six months. During these visits, a predefined subset of investigations will be performed (See Table 1). Further, medical records from routine clinical care will be requested. Our study has been approved by the medical ethical reviewing committee Region Arnhem – Nijmegen (NL64269.091.17; 2017–3911) and is registered at ClinicalTrials.gov (NCT04478981).

**Table 1:** Examinations performed in LAST STRONG study

Outcome domain	Examination	Age	Visit
**Medical history**			
Past	Perinatal period, motor milestones	All	1
Current	Functional abilities, comorbidities, devices, treatments	All	All
**Neurological examination and functional measurements**			
Muscle function	Muscle strength assessment (Medical Research Council, MRC)	≥ 5 years	All
	Hand-Held dynamometry (HHD)	≥ 5 years	All
	The Children’s Hospital of Philadelphia Infant Test of Neuromuscular Disorders (CHOP INTEND)	< 2 years	All
	Hammersmith Infant Neurological Examinations (HINE)	< 2 years	All
	Motor Function Measurement (MFM)-20/320	≥ 2 years	All
	Hammersmith Functional Motor Scale (HFMS); non-ambulant participants only	≥ 2 years	All
	Pediatric Balance Scale (PBS); ambulant participants only	2–15 years	All
	Mini Balance Evaluation Systems Test (miniBEST); ambulant participants only	≥ 16 years	All
	Graded and timed function tests; ambulant participants only		All
	1. 30 s sit to stand, climb 4 stairs, rise from the floor, timed up and go (TUG)	≥ 2 years	
	2. 6-Minute Walk test (6MWT), 10-Meter Walk test (10MWT)	≥ 5 years	
	Functional Ambulation Classification (FAC)	≥ 5 years	All
	Vignos and Brooke scale	≥ 2 years	All
Contractures	Goniometry	≥ 2 years	All
Other	Coordination, gait, reflexes, cranial nerves and facial muscles, dysmorphic features	≥ 2 years	All
**Questionnaires**			
Quality of Life	Pediatric Quality of Life Inventory (PedsQL) (Generic Core Scale, Neuromuscular Module; Child Self-report and/or parent proxy)	2–17 years	All
	Research and Development-36 (RAND36)	≥ 18 years	All
	Individualized Neuromuscular Quality of Life (INQoL)	≥ 18 years	All
Pain	McGill pain questionnaire	≥ 12 years	All
	Wong-Baker Faces Pain rating scale	≥ 2 years	All
Fatigue	Checklist Individual Strength (CIS)	≥ 18 years	All
	Pediatric Quality of Life Inventory (PedsQL) (Multidimensional Fatigue Scale; Child Self-report and/or parent proxy)	2–17 years	All
Activities and participation	ACTIVLIM	≥ 6 years	All
	Impact on Participation and Autonomy (IPA)	≥ 18 years	All
	Egen Klassifikation version 2 (EK2)	≥ 12 years	All
	Borg Rating Scale of Perceived Exertion; prior to and after 6MWT	≥ 5 years	All
**Imaging**			
Muscles	Muscle ultrasound	All	All
	Full body muscle magnetic resonance imaging (MRI)	≥ 10 years	1, 3
Spine	X-ray of total spine (anteroposterior, lateral) and of lumbar spine (flexion and extension))	≥ 2 years	1, 3
Bone density	Dual-energy X-ray absorptiometry (DEXA-)scan	≥ 2 years	1, 3
**Cardiopulmonary assessment**			
Heart	Electrocardiogram	≥ 2 years	1, 3
	Conventional echocardiography	≥ 2 years	1, 3
Lungs	Spirometry (forced expiratory volume in the first second (FEV1), forced vital capacity (FVC), vital capacity (VC), peak cough flow (PCF))	≥ 5 years	All
	Maximum Expiratory Pressure (MEP), Maximum Inspiratory Pressure (MIP) and Sniff Nasal Inspiratory Pressure (SNIP)	≥ 5 years	All
	Ultrasound of the diaphragm	All	All
**Accelerometry**			
GENEActiv	Accelerometry for eight consecutive days	≥ 2 years	All

### Study population

Based on the prevalence reported in previous studies on SELENON-RM and LAMA2-MD, we expect to identify between 20-30 patients in each disease group. Based on our experience in rare diseases, we estimate that around 50% of the patients will participate in the study. We therefore aim to include 10-15 participants in each disease group. Inclusion criteria include a genetic confirmation of SELENON-RM or LAMA2-MD by two recessive pathologic mutations in the *SELENON* or *LAMA2* gene, respectively, or typical clinical and histological alterations combined with genetic confirmation in a first degree relative. Additionally, patients must be willing and able to complete (part of) the measurement protocol in the Radboud university medical center. If patients do not wish or are not able to visit our center, they are offered to participate in this study by sharing medical records, completing questionnaires, and undergoing a medical history and physical examination through home visits, video and/or telephone interview. Exclusion criteria are an insufficient understanding of the Dutch language and the unwillingness of the patient or his/her legal representatives to provide written informed consent for participation in our study.

### Recruitment

**Fig. 1:**
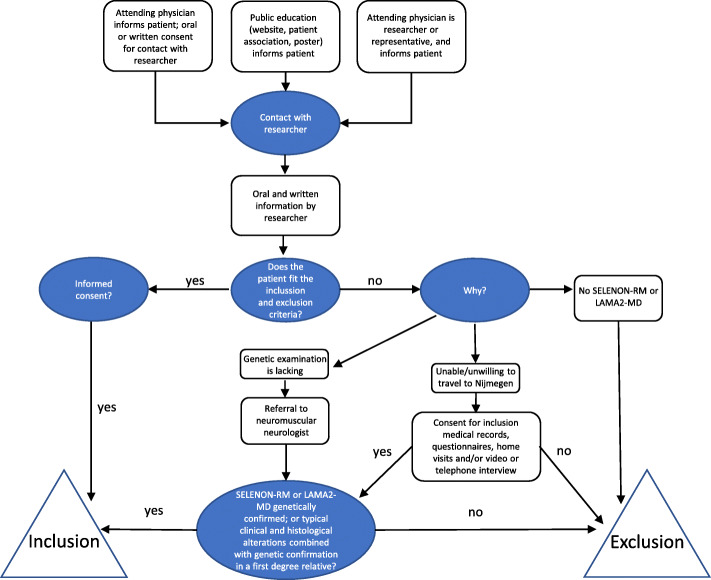
Flow chart of recruitment and inclusion of SELENON-RM or LAMA2-MD patients

Participants will be recruited non-selectively and consecutively in the periods from August 2020 to August 2021 (*See* Fig. 1*)*. In order to estimate the prevalence and to provide data on the complete spectrum of patients with SELENON-RM or LAMA2-MD, we aim to reach all patients in The Netherlands and the Dutch-speaking part of Belgium (Flanders) in our study. All Dutch and Dutch-speaking Belgian (pediatric) neurologists, rehabilitation specialists and clinical geneticists are personally asked about potential participants. Additionally, all patients known at our own neuromuscular center will be personally informed. Moreover, we will directly recruit participants through promotion of our study on patient information days, social media and through patient organizations.

### Demographics

Date of birth, sex, weight (kg), height (m), comorbidity and medication will be recorded.

### Genetics

Upon inclusion in our study, all participants (or a first degree relative) have undergone genetic examination as part of regular diagnostic work-up. The genetics reports, including information on the specific genetic alterations, will be requested. Regarding LAMA2-MD, we expect to mostly include patients harboring the Dutch founder mutation in the *LAMA2* gene (c.5562 + 5G > C). All genetic laboratories in The Netherlands will be contacted to inventory the number of patients diagnosed with SELENON-RM or LAMA2-MD in order to contrast this number against the number of participants in our natural history study.

### Muscle biopsy

Pathological records and stained slides will be requested from participants diagnosed with LAMA2-MD in whom muscle biopsy material was previously taken as part of regular diagnostic work-up. Hereby we aim to classify LAMA2-MD patients as either suffering from a complete or a partial Laminin subunit α2 deficiency.

### Neurological examination and functional measurements

All patients undergo a standard neurological examination by one assessor (KB). Additionally, muscle strength, facial muscle weakness, reflexes, muscle tone, and dysmorphic features are assessed by two independent assessors (KB and CE or NV). Muscle strength (MRC grading scale) will be assessed of the following muscles: neck flexor, neck extensor, sternocleidomastoid, trapezius, deltoid, biceps brachii, triceps brachii, wrist extensor, wrist flexor, finger extensor, finger flexor, finger spreader, iliopsoas, gluteus maximus, quadriceps, hamstrings, foot dorsiflexor, foot plantarflexor, extensor hallucis longus and toe flexor muscles. Further, muscle strength of the following muscles will be measured using a hand-held dynamometer (Citec, CT3002) [38–41]:

1. Neck flexors and neck extensors: sitting upright; head up at 90° from horizontal
2. Elbow flexors and extensors: supine; shoulder adducted, elbow 90° flexed, forearm supinated
3. Knee extensors: sitting upright; knee 90° flexed
4. Foot plantar- and dorsiflexors: supine; foot 90° dorsiflexed
5. Pinch grip: sitting upright; shoulder adducted, elbow 90° flexed, forearm pronated

Additionally, the passive range of motion (PROM) of the elbow, wrist, hip, knee and ankle joints is assessed by a goniometer [42]. Functional measurements include:

1. The Children’s Hospital of Philadelphia Infant Test of Neuromuscular Disorders (CHOP INTEND) (age < 2 years) [43, 44].
  - CHOP INTEND has been shown to be valid for the assessment of motor skills of children below 2 years of age.
2. Hammersmith Infant Neurological Examinations (HINE) (age < 2 years) [45].
  - HINE is designed to be a simple and scorable method for evaluating infants from 2 months to 2 years of age. It includes three sections that assess different aspects of neurologic function, including neurological examination, developmental milestones and behavioral assessment.
3. Motor Function Measure – 20/32 (MFM-20/32) (age ≥ 2 years) [46, 47].
  - Motor function in patients with neuromuscular diseases can be measured with the MFM. The MFM is a scale which consists of 20 or 32 items in three dimensions: D1: standing position and transfers, D2: axial and proximal motor function, D3: distal motor function. MFM-32 is used in adults and in children of 7 years and older. Children with the age of 2 to 7 years will undergo MFM-20.
4. Hammersmith Functional Motor Scale (HFMS) (age ≥ 2 years; non-ambulant participants only) [48, 49].
  - The HFMS was originally developed to assess the physical abilities of children with non-ambulant spinal muscular atrophy. It consists of 20 items that were considered as important to measure the physical functioning of those patients.
5. Pediatric Balance Scale (PBS) (pediatric patients aged 2–15 years; ambulant participants only) [50].
  - The PBS is a modified version of the Berg Balance Scale that is used to assess functional balance skills in school-aged children with mild to moderate motor impairments.
6. Mini Balance Evaluation Systems Test (miniBEST) (age ≥ 16 years; ambulant participants only) [51].
  - The miniBEST evaluates balance control by scoring of exercises that belong to one of the following categories: anticipatory postural changes, reactive postural control, sensory orientation and walking.
7. Graded and timed function tests (age ≥ 2 years: 30 s sit to stand, climb 4 stairs, rise from the floor, timed up and go (TUG); age ≥ 5 years: 6-Minute Walk test, 10-Meter Walk test; ambulant participants only) [52–54].
8. Functional Ambulation Classification (FAC) (age ≥ 5 years) [55].
  - The FAC assesses functional ambulation in patients.
9. Brooke and Vignos scale (age ≥ 2 years) [56–58].
  - The Brooke and Vignos scales provide ordinal-level data to assess the upper and lower extremity functions, respectively.

Neurological examination and functional measurements will be performed at all four visits.

### Questionnaires

Each visit, patients and/or their parent(s) will be asked to complete age-adapted questionnaires on quality of life, pain, fatigue, and activities and participation,. These questionnaires include:

1. Pediatric Quality of Life Inventory (PedsQL) (Generic Core Scale, Neuromuscular Module, Multidimensional Fatigue Scale; Child Self-report and/or parent proxy) (pediatric patients 2–17 years) [59–61].
  - The PedsQL Generic Core Scale consists of 23 questions in four domains: Physical, Emotional, Social, and School Functioning. It has been translated and subsequently validated into many languages, including Dutch.
  - The PedsQL Neuromuscular Module consists of 25 questions in three domains: Neuromuscular disease, Communication and Family resources
  - The PedsQL Multidimensional Fatigue Scale assesses subjective fatigue in three domains, namely General Fatigue Scale, Sleep/Rest Fatigue Scale, and Cognitive Fatigue Scale.
2. Research and Development-36 (RAND36) (age ≥ 18 years) [62].
  - Measure for quality of life with 36 items.
3. Individualized Neuromuscular Quality of Life (INQoL) (age ≥ 18 years) [63].
  - The INQoL is a validated muscle disease specific measure of quality of life, which can be used for individuals or large samples.
4. McGill pain questionnaire (age ≥ 12 years) [64].
  - Questionnaire in which the location, level and characteristics of pain are assessed.
5. Wong-Baker Faces Pain rating scale (age ≥ 2 years) [65].
  - The Wong-Baker Faces Pain Scale was originally created for children to help them communicate about their pain.
6. Checklist Individual Strength (CIS) (age ≥ 18 years) [66, 67].
  - The CIS is a questionnaire rating four subscales: subjective tiredness, concentration, motivation and physical activity. It consists of 20 items on a seven-point scale.
7. ACTIVLIM (age ≥ 6 years) [68].
  - Questionnaire to assess the ability to perform 22 activities of daily life on a three-point scale from impossible to easy.
8. Impact on Participation and Autonomy (IPA) (age ≥ 18 years) [69].
  - Questionnaire about participation and autonomy in daily life.
9. Egen Klassifikation version 2 (EK2) (age ≥ 12 years and sufficient understanding of the English language) [70].
  - The EK2 is a questionnaire that was designed to measure functional ability of activities in daily living in non-ambulant Duchenne muscular dystrophy patients. This questionnaire is available in English. Therefore, only patients who have a sufficient understanding of the English language will be asked to complete this questionnaire.
10. Borg Rating Scale of Perceived Exertion (Borg RPE scale) (age ≥ 5 years) [71].
  - The Borg RPE scale is used to assess physical activity intensity level. In the LAST STRONG study participants are asked to assess their psychical sensations prior to and after the 6MWT.

### Imaging

At all four visits, muscle thickness and muscle echogenicity (quantitative grayscale analysis and Heckmatt ultrasound score) of a subset of bilateral muscles (See Table 2) will be assessed by muscle ultrasound using an Esaote MyLabTwice ultrasound scanner (Esaote SpA, Genoa, Italy) with an 3–13 MHz broadband linear transducer and a 53-mm footprint, adhering to a strictly defined and fixed measurement protocol [72–78]. To measure muscle echogenicity, the mean grayscale level within a manually selected region of interest (ROI) in the ultrasound image is calculated using an in-house developed software package in MATLAB (version 2013b, Mathworks, Natick, MA, USA). The muscle echogenicity and thickness are standardized by calculating their z-score: [[measured grayscale level – predicted grayscale level] / standard deviation grayscale]. The predicted grayscale level is calculated using a reference equation including age, length, sex and weight [78, 79]. In addition, all ultrasound images will be visually evaluated using the semi-quantitative Heckmatt grading scale [80]. At visit 1 (t = 0) and visit 3 (t = 12 months), muscle features are additionally qualitatively and (semi-)quantitatively described through full body muscle MRI (1,5 Tesla, Siemens, Erlangen, Germany) in accordance with our locally developed scanning protocol including Dixon vibe and Short-TI Inversion Recovery (STIR) images [81–84]. In accordance with previous studies on quantitative assessment of muscle MRI, the water and fat image of the Dixon sequence will be used to create a fat fraction map using MATLAB according to the following equation: Fat/(Fat + Water) [84]. The fat fraction map will be used to draw ROI (region of interest) per muscle using ImageJ software (ImageJ 1.47v, National Institutes of Health, USA). ROI will be drawn at predefined localization using the localizer sequences. All drawn ROI will be checked by a second clinician. Muscle cross-sectional area and fat fractions will be calculated per ROI. The qualitative and semi-quantitative assessment will be performed by two independent assessors. It will include assessment of a predefined subset of bilateral muscles (See Table 3), including relative reduction of muscle volume (0: no reduction, 1: mild, reduction of < 30%, 2: moderate, reduction of 30–60%, 3: severe, reduction of > 60%, 4: non-identifiable muscle), fatty infiltration (Modified Mercuri scale [85–87]), inflammation (absent/present, plus pattern: patchy, diffuse, peripheral/perifascial, central, other), and fibrosis (absent/present, plus pattern: fascial, intramuscular or other). A MRI will only be performed in patients of 10 years or older who are able to lie still for 45 min without respiratory equipment.

**Table 2:** Muscle ultrasound in a large subset of bilateral skeletal muscles

Muscle	Point of measurement
M. Temporalis	Parallel to the oculus (above os zygomaticum)
M. Sternocleidomastoid	1/2 line lobulus auriculae to clavicula
M. Biceps brachii	2/3 line acromion – elbow fossa
M. Flexor carpi radialis	1/3 line elbow fossa – caput radii
M. Erector spinae thoracalis	At the level of seventh thoracic vertebrae
M. Erector spinae lumbalis	At the level of third lumbar vertebrae
M. Rectus abdominis	2 cm above umbilicus
M. Biceps femoris	1/2 line gluteal sulcus – popliteal fossa
M. Rectus femoris	1/2 line spina iliaca – upper edge of patella
M. Vastus lateralis	2/3 lateral line spina iliaca – upper edge of patella
M. Gastrocnemius - caput mediale	1/3 line popliteal fossa – medial malleolus
M. Soleus	The place where gastrocnemius disappears and the fibula appears
M. Tibialis anterior	1/3 line lower edge patella – lateral malleolus

**Table 3:** Qualitative and semi-quantitative assessment of a large subset of bilateral skeletal muscles through MRI

Lower extremity and pelvic girdle	Upper extremity, shoulder girdle and trunk
m. Extensor digitorum longus	m. Sternocleidmastoideus
m. Flexor digitorum longus	Neck flexor muscles
m. Gastrocnemius medialis	Neck extensor muscles
m. Gastrocnemius lateralis	m. Levator scapulae
m. Soleus	m. Longus colli
m. Tibialis posterior	m. Latissimus dorsi
m. Tibialis anterior	m. Trapezius
m. Vastus intermedius	m. Deltoideus
m. Vastus medialis	m. Rotatorcuff muscles (Subscapularis)
m. Vastus lateralis	m. Pectoralis major
m. Rectus femoris	m. Pectoralis minor
m. Biceps femoris - short head	m. Serratus anterior
m. Biceps femoris - long head	Anterior arm compartment (m. biceps brachii)
m. Semitendinosus	Posterior arm compartment
m. Semimembranosus	Anterior forearm compartment (m. Flexor carpi radialis)
m. Adductor longus	Posterior forearm compartment
m. Adductor brevis	m. Intercostales
m. Adductor magnus	m. Erector thoracalis spinae
m. Gracilis	m. Erector lumbalis spinae
m. Sartorius	m. Quadratus lumborum
m. Tensor fascia latae	Abdominal belt muscles (m. Rectus abdominis)
m. Quadratus femoris	
m. Gluteus maximus	**Head**
m. Gluteus medius	m. Temporalis
m. Gluteus minimus	m. Masseter
m. Psoas	m. pterygoideus medialis
m. Iliacus	m. pterygoideus lateralis
m. Piriformis	Tongue
m. Obturator internus	
m. Obturator externus	
m. Perineal muscles	
m. Pectineus	

At visit 1 (t = 0) and visit 3 (t = 12 months), an X-ray will be performed to assess deformities of the spine, including Cobb’s angle (spinal curvature), pelvic obliquity, coronal and sagittal balance and lumbar flexion and extension [88, 89]. In order to assess bone density, a DEXA-scan of the right femoral neck and lumbar spine, including a vertebral fracture assessment, will be performed at visit 1 (t = 0) and visit 3 (t = 12 months) by using the Hologic Discovery A Horizon DXA System (S/N 303053 M).

### Cardiac assessment

In order to describe the prevalence and progression of cardiac comorbidities and the need for routine cardiac assessment, patients (age ≥ 2 years) will undergo electrocardiogram and conventional transthoracic echocardiography (TTE) with speckle tracking and Tissue Doppler Imaging (TDI) at visit 1 (t = 0) and visit 3 (t = 12 months). All investigations will be performed by EACVI TTE certified sonographers using commercially available ultrasound systems (Affiniti70 General, Philips Healthcare, Best, the Netherlands for adult participants; or Vivid E9 or Vivid E95, GE Healthcare Ultrasound, Horten, Norway for pediatric participants). Offline analysis will be performed using dedicated software (AGFA Enterprise Imaging Cardiology version 8.1.2, AGFA HealthCare, Mortsel, Belgium). Global Longitudinal strain (GLS) will be measured using speckle tracking echocardiography on a three beats acquisition with a frame rate > 60 frames/sec. All measurements will be done according to the EACVI recommendations for cardiac chamber quantification [90].

### Pulmonary function

At all visits, patients (age ≥ 5 years) will undergo spirometry (forced expiratory volume in the first second (FEV1), forced vital capacity (FVC), vital capacity (VC), peak cough flow (PCF)) in upright and supine position (SpiroUSB, Vyaire Medical connected to PC Spirometry software, Spida CareFusion 2.3.0.10 for Windows 7). Additionally, patients will undergo Maximum Expiratory Pressure (MEP), Maximum Inspiratory Pressure (MIP) and Sniff Nasal Inspiratory Pressure (SNIP) assessment in upright position (Micro RPM, Micro Medical, CareFusion, United Kingdom) [91]. Diaphragm ultrasound (MyLabTwice, Esaote SpA, Genoa, Italy) will be performed to assess diaphragm thickness (end expiratory and maximum inspiratory thickness) and thickening, and diaphragm echogenicity [92–94].

### Accelerometry

After all visits, patients (age ≥ 2 years) are asked to wear an accelerometer (GENEActiv Original, Activinsights Ltd) for eight consecutive days. In the same time period, patients or their parents are asked to fill in a diary with their major activities, including sleeping hours, physical exercise, work and school. The GENEActiv is a tri-axial, wrist-worn accelerometer that will be set to measure at 87,5 Hz sampling [95–97]. The raw data will be converted into 1-s epochs by using the GENEActiv Software (v.3.3, 2019). We will use the gravity subtracted sum of vector magnitudes (SVMgs) as the activity measure. The SVMgs will be measured using the following equation: SVMgs = ∑√(x^2^ + y^2^ + z^2^) -1 g. All data will be subsequently analyzed using MATLAB R2018a Update 4 (9.4.0.902940) for windows. A large subset of parameters will be addressed, including total activity (counts/day), the percentage of sedentary, light, moderate or vigorous activity, and total activity during sleep.

### Statistical methods

Due to the explorative character of our study, we will use descriptive statistics (mean, median, SD, 95%-CI) in order to summarize our data. Further, Spearman’s correlation analysis and non-parametrical testing will be used to test the correlation between key parameters (i.e. age, ancillary investigations). Further, parameters will be corrected for genetic differences and partial or complete laminin subunit α2 deficiency. If reference values are available from literature, we will check for overlapping confidence intervals. In order to assess disease progression between subsequent measuring moments, we will perform the Wilcoxon signed-rank test (nonparametric continuous paired data) and the McNemar’s test (categorial paired data). Multiple linear regressions will be used to explore the relationship between potential disease modifying variables and disease severity. Linear mixed models will be applied for analysis of differences in disease progression. Further, we will correct for multiple testing. We regard *p* < 0,05 as statistically significant. The Statistical Package for the Social Sciences (SPSS version 25, IBM, Armonk, New York) will be used to conduct all statistical analyses.

### Data collection

All data-management and data-monitoring will be performed within the Castor software (Version 2021) through direct entry or indirect entry via our electronic patient system (Epic, version May 2020).

### Selection of outcome measures for future clinical trials

The results of our natural history study on both SELENON-RM and LAMA2-MD will feed an international key-opinion-leader workshop in which the in- and exclusion criteria, follow-up frequency and outcome measures for the international natural history studies will be discussed. When the results of preclinical and clinical work on promising new therapies continue to be encouraging, a clinical trial will be initiated.

### Time plan

Patients will be included between August 2020 and August 2021 and will be followed for 1,5 year. The last visit of the last included patient is expected to take place in the beginning of 2023.

## Discussion

Here we present the design of the LAST STRONG study, an extensive natural history study in an unselected cohort of Dutch-speaking SELENON-RM and LAMA2-MD patients (both children and adults) that includes a broad plethora of clinical and functional outcome measures. This enables us to assess the clinical spectrum of patients diagnosed with SELENON-RM or LAMA2-MD. Hereby, we aim to fulfill our primary objectives namely, 1. to assess 1.5-year natural history in patients with SELENON-RM or LAMA2-MD; and 2. to select relevant and sensitive clinical and functional outcome measures to reach trial readiness. In addition, our insights will be vital for reaching our secondary objectives, including making a prevalence estimation, establishing a well-characterized baseline cohort and providing adequate symptomatic management leading to improved clinical care. We propose elaborate qualitative and quantitative measurements adapted to the age and functional abilities of the participant. The structured approach enables us to give an explorative, full-spectrum clinical description of patients diagnosed with SELENON-RM or LAMA2-MD. Further, there is a well-organized health system with full access to (pediatric) neurology and rehabilitation for every patient. Our center also has a longstanding history of patient recruitment for scientific research. Altogether, this is expected to reduce selection bias. Further, as a tertiary referral center for neuromuscular diseases, we are widely experienced with the clinical care and the conduction of studies in patients with rare neuromuscular diseases. Finally, all participants can reach our study center within a three-hour travel by car, which enables us to execute a population based, nationwide study. A consequence of our natural history study might be that the most severely affected patients may be less likely to participate in our study. We expect to overcome this problem to some extent by requesting all available medical records, sending out questionnaires and performing a medical history by video call/telephone or home visits for all patients that are not able or do not wish to visit our center, provided that written informed consent is given. Due to nationwide travel restrictions and local hospital measures related to the COVID-19 pandemic, Belgian patients are limited in their participation in our study.

The rarity of SELENON-RM and LAMA2-MD will result in a relatively small sample size of Dutch-speaking patients, which limits our possibilities to verify correlations or differences in subgroups. Consequently, we aim to contribute to international collaborations in order to enlarge the availability of natural history data.

The importance to reach trial readiness is warranted by the recent development of promising new treatment strategies for SELENON-RM and LAMA2-MD. Outcome measures need to be reliable and specified per age and disease severity for an adequate measurement of disease progression. If these outcome measures are not reliable, possible positive effects of future treatments in clinical trials will remain to be unrevealed. Reliable outcome measures are thus required in order to start clinical trials.

Further, deep phenotyping of our cohort provides us with valuable information on disease characteristics, enabling us to fulfil one of our secondary objectives, i.e. improving clinical care. For example, participants in whom clinically significant (co)morbidities are found during our study, are referred to the appropriate medical specialists. Moreover, we can take these (co)morbidities into account for routine clinical care of patients with SELENON-RM or LAMA2-MD that are not participating in our natural history study.

In our study, we do not take into account the selection of serum or urine biomarkers in order to limit the burden for patients participating in this study. However, in a study by Bharucha-Goebel et al. serum samples of patients with LAMA2-MD were analyzed for biomarkers. Proteins that were found to be altered, primarily consisted of cytokines and proteins involved in development and cell adhesion. Of the proteins decreased, most were cytokines, growth factors, and protease inhibitors [98]. We recommend to take serum or urine biomarkers into account in future studies.

## Conclusion

The LAST STRONG study is expected to provide natural history data, which can be used for the selection of relevant clinical and functional outcome measures in order to reach trial readiness in SELONON-RM and LAMA2-MD patients. Further, our study aims to optimize clinical management in patients diagnosed with SELENON-RM or LAMA2-MD.

## Data Availability

The datasets generated during the current study will be available in the Donders Repository (https://data.donders.ru.nl).

## Abbreviations

Borg RPE: Borg Rating Scale of Perceived Exertion
CHOP INTEND: The Children’s Hospital of Philadelphia Infant Test of Neuromuscular Disorders
CIS: Checklist Individual Strength
DEXA-scan: Dual Energy X-ray absorptiometry
EK2: Egen Klassifikation version 2
FAC: Functional Ambulation Classification
FEV1: Forced expiratory volume in the first second
FVC: Forced vital capacity
HFMS: Hammersmith functional motor scale
HHD: Hand-Held dynamometry
HINE: Hammersmith neurological examinations
INQoL: Individualized Neuromuscular Quality of Life
IPA: Impact on Participation and Autonomy
LAMA2-MD: LAMA2-related muscular dystrophy
MDC1A: Merosin-deficient congenital muscular dystrophy type 1A
MEP: Maximum Expiratory Pressure
MFM-20/32: Motor function measure – 20/32
miniBEST: Mini Balance Evaluation Systems Test
MIP: Maximum Inspiratory Pressure
MRC: Medical Research Council
MRI: Magnetic resonance imaging
PBS: Pediatric Balance Scale
PCF: Peak cough flow
PedsQL: Pediatric Quality of Life Inventory
PROM: Passive range of motion
RAND-36: Reasearch and Development-36
ROI: Region of interest
SELENON-RM: SELENON-related myopathy
SNIP: Sniff Nasal Inspiratory Pressure
STIR: Short-TI Inversion Recovery
SVMgs: The gravity subtracted sum of vector magnitudes
TDI: Tissue doppler imaging
TTE: Transthoracic echocardiography
VC: Vital capacity
6MWT: 6-Minute Walk Test
10MWT: 10-Meter Walk Test
